# Automated Detection Algorithm for Mesoscale Heated Regions in TWINS Ion Temperature Maps

**DOI:** 10.1029/2022JA030464

**Published:** 2022-09-23

**Authors:** A. M. Keesee, R. Katus, J. Tibbetts, J. Liu, X. Zhang, K. A. Sorathia

**Affiliations:** ^1^ Department of Physics and Astronomy University of New Hampshire Durham NH USA; ^2^ Space Science Center University of New Hampshire Durham NH USA; ^3^ Department of Mathematics Eastern Michigan University Ypsilanti MI USA; ^4^ Department of Earth, Planetary, and Space Sciences Institute of Geophysics and Planetary Physics University of California Los Angeles CA USA; ^5^ The Johns Hopkins University Applied Physics Laboratory Laurel MD USA

**Keywords:** geomagnetic storms, energetic neutral atom imaging, magnetotail, plasma sheet, dipolarizing flux bundles, substorms

## Abstract

Earth's magnetotail plays a critical role in the dynamics of the magnetosphere, particularly during intervals of geomagnetic activity. To improve our understanding of the ion dynamics in this region, energetic neutral atom (ENA) imaging can provide global measurements to place in situ measurements in context and validate simulations. The NASA Two Wide‐angle Imaging Neutral‐atom Spectrometers mission provided near‐continuous observations using ENA imagers. ENA data can be used to calculate maps of equatorial ion temperatures that often show observations of regions of enhanced temperatures associated with phenomena in the magnetotail such as magnetic reconnection and narrow flow channels. We present an algorithm that can be used to search through a collection of these maps to identify intervals with such enhancements for further study. The algorithm results are validated against two sets of related phenomena: (a) a database of dipolarizing flux bundle (DFB) measurements from THEMIS and (b) a list of substorm onsets from SuperMAG. We demonstrate that the algorithm is very good at identifying intervals when there are DFB measurements or substorm onsets as long as there sufficient ENA data. We discuss some potential scientific studies that can result from use of the algorithm. We also show a preliminary application of the algorithm to simulation output to demonstrate the usefulness for other datasets, facilitate comparative studies, and introduce a new method for model validation.

## Key Points

- An algorithm has been developed to identify regions of increased ion temperatures in maps calculated from Two Wide‐angle Imaging Neutral‐atom Spectrometers energetic neutral atom data
- The algorithm is validated against a list of dipolarizing flux bundle measurements and a list of substorm onsets
- The algorithm will be useful for future case and statistical studies of mesoscale phenomena as well as model validation

## 1 Introduction

Magnetotail dynamics are a critical element of understanding and predicting what happens in the magnetosphere during geomagnetic storms and substorms. Magnetic reconnection in the magnetotail energizes ions and electrons and accelerates them Earthward. These energized particles have been found to travel in narrow channels in a bursty manner (Angelopoulos et al., 1992). The mesoscale features associated with these narrow channels have been called bursty bulk flows (BBFs) (Angelopoulos et al., 1992), dipolarizing flux bundles (DFBs) (Liu et al., 2014), and bubbles (Pontius & Wolf, 1990), depending upon the measurements being used to describe the features. However, there are many open questions about the processes related to these features in the magnetotail. For example, it is unclear whether all Earthward traveling particles are the result of reconnection. Fu et al. (2013) reported observations of a dipolarization front (DF) associated with transient reconnection, but argued that DFs observed in other studies (e.g., Nakamura et al., 2013; Runov et al., 2012) demonstrated evidence of other mechanisms such as jet braking and spontaneous formation. Another question is where and how these particles enter the inner magnetosphere and how much they contribute to the ring current and radiation belts. The currents that develop during substorm expansion, known as the substorm current wedge, may be made up of smaller “wedgelets” (e.g., Nakamura et al., 2005; Rostoker, 1998). Liu et al. (2013) found that the currents associated with a DFB can serve as such a wedgelet, and Liu et al. (2015) found that the collective effect of a few of them is sufficient to serve as the large scale substorm current wedge. These mesoscale features have been shown to be an important contributor to the transport of energetic particles to the inner magnetosphere, though this has been difficult to quantify (e.g., Gkioulidou et al., 2014).

The limited spatial and temporal information from in situ satellites makes answering these questions very challenging. While simulations can provide some global information about these processes, simultaneous measurements across the magnetotail are needed to provide an actual picture of these phenomena for validation and comparative studies. Sorathia, Ohtani, et al. (2021) and Sorathia, Michael, et al. (2021) used a combination of global MHD and test particle simulations to study ion transport to the inner magnetosphere and found mesoscale structures to be a critical element. While their results agreed with statistical studies from in situ satellites, it would be helpful to compare single events which can be more challenging due to lack of satellite coverage.

Energetic neutral atom (ENA) imaging can provide global measurements to help address these open questions. A technique to calculate ion temperature maps from ENA data has been developed (Keesee et al., 2008; Scime et al., 2002) and used to study mesoscale phenomena in the magnetotail. These maps have been used to observe dawnward deflection of the flow channels (Keesee et al., 2014), demonstrate that some ion heating processes are missing from global simulations (Keesee et al., 2021), and show that global magnetosphere reconfigurations change the way the magnetotail and ionosphere are coupled (Adewuyi et al., 2021). To help answer the open questions described above, more statistical and case studies are needed that take advantage of the information provided by these maps. To support this effort, ion temperature maps calculated from the Two Wide‐angle Imaging Neutral‐atom Spectrometers (TWINS) ENA imagers with 10‐min cadence were created for storms that occurred 2009–2017 in the database described by Keesee et al. (2020).

Additionally, an algorithm has been developed to identify regions of increased ion temperatures in these maps, which are associated with mesoscale phenomena in the magnetotail. In this paper, we present the details of the algorithm, present examples of intervals selected by the algorithm, and perform a validation to improve our understanding of phenomena associated with the identified regions. We also present a preliminary application of the algorithm to simulation output that will enable a new method for model validation and data‐model comparisons of mesoscale phenomena.

## 2 TWINS Data and Ion Temperature Maps

The TWINS mission is a NASA mission of opportunity on two satellites in high‐inclination Molniya orbits (McComas et al., 2009). Each satellite has an ENA imager providing remote detection of ions in the magnetosphere via charge exchange with the neutral exosphere. Data are available for 2009–2019.

The TWINS ENA data have been used to calculate ion temperature maps of the inner magnetosphere and magnetotail by projecting the data along the instrument line of sight to a 160 × 160 grid with 0.5*R*_*E*_ × 0.5*R*_*E*_ bins in the GSM equatorial plane and fitting a Maxwellian in each grid bin (Keesee et al., 2011). This spatial resolution is high enough for imaging DFBs whose smallest dimension in the equatorial plane is typically 1–3 *R*_*E*_ wide (e.g., Liu et al., 2013). A database of these maps at 10‐min cadence is available on CDAWeb and described by Keesee et al. (2020). Initial tests of the algorithm were run on maps from the database, and visual inspection was used to refine the algorithm parameters. To validate the algorithm, we ran select storms at a 3‐sweep (∼4 min) cadence chosen to overlap with the validation data sets described below. To simplify the set of ion temperature maps calculated from the two separate satellites (TWINS 1 and TWINS 2), we created a combined set of maps. For intervals when only one satellite was available, the map from that satellite is used. When data were available from both satellites, an averaged map was created using an averaged temperature for any map bins where both satellites had data but keeping the single satellite temperature for any bins that had data from only one as described by Keesee et al. (2020).

## 3 Algorithm

An automated detection algorithm has been developed to determine which ion temperature maps have regions of increased temperatures. The goal of the algorithm is to aid in identifying intervals of interest with mesoscale features in the magnetotail. This automated detection method follows a simple algorithm but is very effective. We note that some of the parameters described here were adjusted and compared during the validation, described below, to select optimal values. The parameters could be varied, but the examples presented in this paper use the values stated in this section. The steps are as follows:

Step 0. Smooth the temperature map using a cubic spline interpolation to scale the image from 160 × 160 to 320 × 320 bins and then performing an average of a 9‐bins square surrounding each bins. This averaged value is then used for each bin in the 160 × 160 grid.

**Figure 1:**
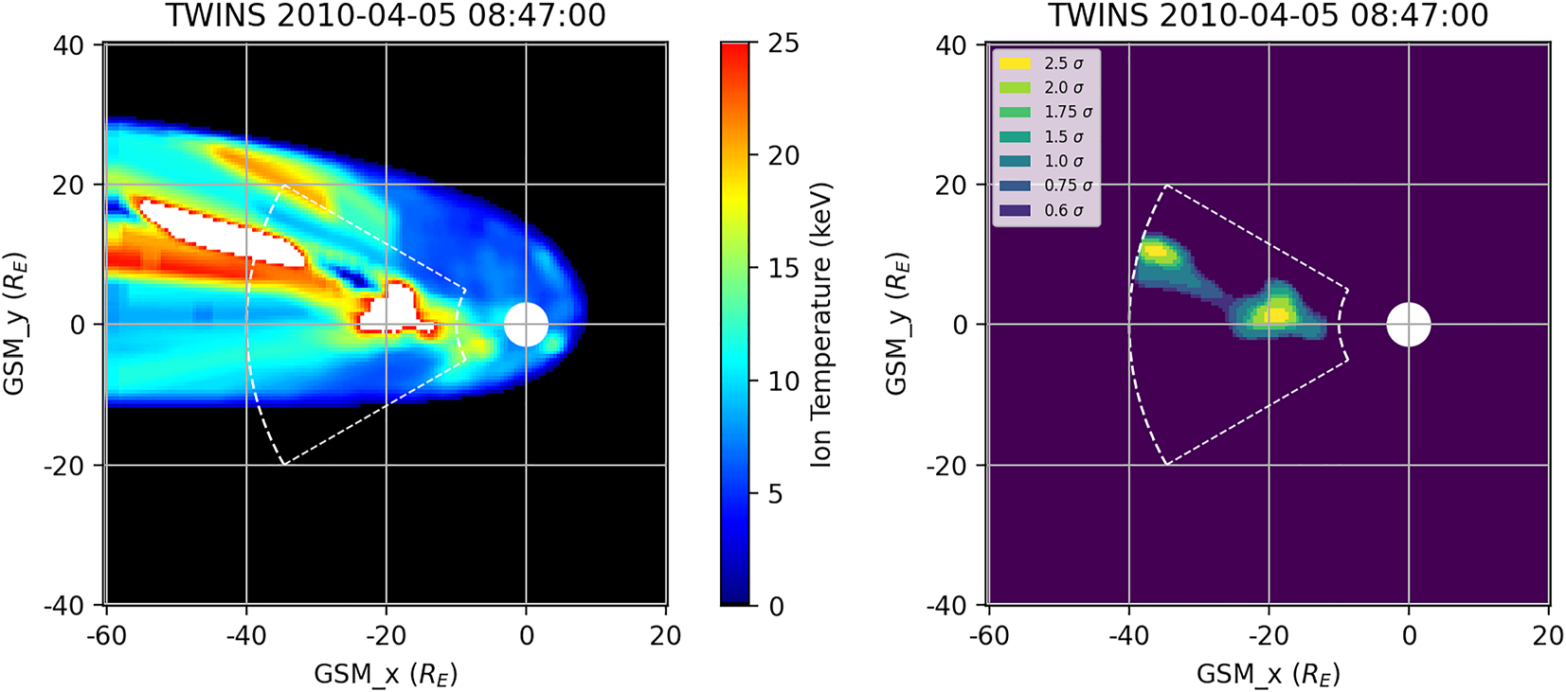
(left) Ion temperature map derived from Two Wide‐angle Imaging Neutral‐atom Spectrometers for 5 April 2010 at 08:47 UT. Dashed outline indicates plasma sheet area searched by algorithm. (right) *Z*‐score map of regions identified from the temperature map using the algorithm. White circle of radius 3*R*_*E*_ is centered at Earth.

Step 1. Define the area to search, assuming the mesoscale features will occur in the plasma sheet. The plasma sheet area searched is contained within the maximum limits of −40*R*_*E*_ < *r* < −10*R*_*E*_ and MLT ≥ 22 hr or MLT ≤ 2 hr, though we tried several versions with different limits. This results in a “baseball outfield” shape (See Figure 1). The values selected include the typical near‐Earth neutral line location along with locations where BBFs are typically observed. Extending *r* or MLT beyond these values includes areas with reduced instrument sensitivity and spatial resolution. Reducing the inner limit to *r* < −5*R*_*E*_ incorporates more inner magnetosphere regions where the LOS projection to the equatorial plane is less reliable. The comparison with the SuperMAG substorm database was better for the *r* < −10*R*_*E*_ limit, indicating regions located at *r* > −10*R*_*E*_ are not all associated with the magnetotail phenomena that we plan to focus on.

Step 2. Designate acceptable data to search. When the ion temperature maps are calculated, spatial bins without a reasonable calculated temperature value are set to a negative constant. Thus, we search only bins with 0 < *T* < 50 keV.

Step 3. Set a minimum condition for the maximum ion temperature in the search area. We do not search the ion temperature map if the maximum ion temperature in the predefined search area is less than 8 keV. During tests of this method, we found these maps to have minimal spatial variation in temperature, so this step reduces unnecessary processing time.

Step 4. Determine bins with ion temperature greater than 2.5 standard deviations above the average ion temperature in the predefined search area. These points define both the tail of the distribution and the peak in variance. We also mark bins with 0.6, 0.75, 1, 1.5, 1.75, and 2 standard deviations above the mean (*z*‐score) to enable further study of their characteristics.

Step 5. Discard selections below a minimum width and minimum size. Regions with less than two continuous bins (each bins is 0.5*R*_*E*_ × 0.5*R*_*E*_) in the *x*‐direction or with less than a total of 25 bins are dropped. This condition removes any random increases that are likely too small to be mesoscale structures. We note that no additional requirements are placed here, for example, the shape of the regions is not predetermined.

Step 6. All intervals with at least one region that matches the criteria are flagged and a *z*‐score (number of standard deviations above the mean) map is created. For intervals with more than one region, separate *z*‐score maps for each region are also created.

A data coverage quality flag is assigned to each interval, indicating the amount of spatial bins within the defined plasma sheet area that are populated. This flag is set to 5 for >95% coverage, 4 for >90%, 3 for >75%, 2 for >50%, 1 for >25%, and 0 for less.

A result found using this algorithm is shown in Figure 1 for 08:47 UT on 5 April 2010. The smoothed (after Step 0) TWINS ion temperature map is shown in the left panel with the “baseball outfield” shape indicating the area searched by the algorithm. The right panel shows the identified regions of increased ion temperatures detected using the automated algorithm, plotted as *z*‐scores. The varying colors in Figure 1 (right panel) indicate the varying values above the mean as described in Step 4. We note that there are two regions identified in this interval.

## 4 Validation

The detection algorithm was developed over several iterations of selecting parameters, considering the results by manually looking at the temperature maps for intervals with and without identified regions, and comparing with two independent datasets using a modified binary event analysis. The modified binary analysis involved determining whether or not (hit or miss) the algorithm identified a region within a set time interval of an identified event in the given data set. The first comparison, using a SuperMAG database of substorm onsets (Forsyth et al., 2015), assumes that the regions of increased ion temperatures detected by the algorithm are associated with substorm activity. The second method, using a list of DFB from THEMIS (Liu et al., 2013; Zhang et al., 2019), is a comparison to local measurements in the magnetotail. In addition to binary analysis with the DFB list, the location of the DFBs were overlaid on the maps to conduct a visual inspection of the spatial correlation between the identified regions and the THEMIS locations. These comparisons were used to adjust the parameters described in Section 3. An example parameter space comparison for −30*R*_*E*_ < *r* < −10*R*_*E*_ and −40*R*_*E*_ < *r* < −10*R*_*E*_ as well as 2*σ* versus 2.5*σ* is shown in Table S1. We chose to use the limit of *r* > −40*R*_*E*_ due to the better hit‐miss ratio with the DFB list (69% hits vs. 91% hits, respectively). However, for the *z*‐score threshold, we chose 2.5*σ* even though the hit‐miss ratio was not as good because the lower *z*‐score resulted in significantly more intervals with identified regions. Since there is not enough satellite coverage to quantify false positives, we chose to be more conservative in our selection. We did a similar comparison of various MLT ranges with the SuperMAG database and found that MLT ≥ 22 hr or MLT ≤ 2 hr yielded the best results.

We also considered whether the algorithm would detect regions that had been identified in previous studies using non‐automated methods. TWINS ion temperature maps during the Galaxy‐15 substorm were calculated and analyzed by Keesee et al. (2014). Multiple intervals were identified by the algorithm during this event that had very intense activity, and one of the identified intervals is shown in Figure 1. We also ran the algorithm on the 3 August 2016 storm selected for study based on MMS data and analyzed by Keesee et al. (2021), showing substorm activity around 5:40 UT. We found that the selection of these intervals by the algorithm was highly dependent on the selected parameters due to the maximum temperature of the area being lower than many other identified intervals (∼10 keV compared to ∼25 keV). A key parameter that resulted in identification of these intervals was the smoothing window size (Step 0). Thus, we selected this parameter based on the inclusion of the interval, shown in Figure S1 in Supporting Information S1 (note that the color scale has a maximum of 10 keV in contrast to the other figures). Thus, we point out that (a) no automated algorithm will be perfect in detecting all and only potentially interesting intervals, so any studies conducted using the resulting intervals must consider that caveat and (b) the parameters of the algorithm can and should be adjusted when considering analyzing varying sets of temperature maps (e.g., different time cadences).

### 4.1 SuperMAG Substorm Database

The first method used to validate the detection algorithm involved a comparison to a substorm database available on the SuperMAG website. Several databases are available, but we focused on the Forsyth et al. (2015) database because it included more substorm onsets with which to compare. We selected 11 storms of varying strength (based on minimum Dst) that had substorm activity (indicated by increases in the AE index). The storms used are shown in Table S2 in Supporting Information S1.

The identification of intervals was compared with the substorm onset list using the modified binary event analysis. The full table of interval comparison data is in Table S3. For each substorm time in the database, *t*_0_, we searched for TWINS intervals in a window from *t*_0_ − 30 min to *t*_0_ + 15 min for an identified region. The results are shown in Table 1, where we indicate the number of substorm events for which TWINS data were available within the window (TWINS Avail), the number for which a region was identified by the algorithm (TWINS Found), and the number for which TWINS data were available but none of the intervals within the window contained an identified region (TWINS Missed). The percentages of *missed* and *found* are also calculated. This calculation is also separated by data coverage quality flag (described in Section 3) to get an idea of whether the *missed* intervals result from lack of data coverage. This certainly appears to be the case because the percentage *found* increases with increasing data coverage. The results demonstrate a resounding success of the algorithm to identify a region in the TWINS maps when there is a substorm as identified from SuperMAG. Thus, if there is a substorm, we can expect to be able to find a region of increased ion temperatures in the TWINS data if we have sufficient data coverage of the plasma sheet area.

**Table 1:** Modified Binary Analysis of Two Wide‐Angle Imaging Neutral‐Atom Spectrometers Algorithm Compared to SuperMAG Substorm Database

	All data	qf ≥ 1	qf ≥ 2	qf ≥ 3	qf ≥ 4	qf = 5
TWINS avail	29	23	20	14	11	9
TWINS found	24	20	18	13	11	9
TWINS missed	5	3	2	1	0	0
Missed %	17%	13%	10%	7%	0%	0%
Found %	83%	89%	90%	93%	100%	100%

We also did an analysis of which TWINS intervals identified by the algorithm were associated with a substorm (Table S4). For each TWINS interval time in the list, *t*_0_, we searched for a substorm in a window from *t*_0_ − 15 min to *t*_0_ + 30 min. Every storm in Table S2 in Supporting Information S1 had at least one match, except for the 4 November 2015 storm. We note that the 4 November 2015 storm was atypical and the NOAA Space Weather Prediction Center indicated that the minor storm was caused by a high speed stream (NOAA Weekly, 2015‐prf2097). The time of substorm was distributed fairly evenly across pre‐storm, sudden storm commencement, main phase, and recovery phase. There were a lot more TWINS intervals with identified regions than there were substorms, so we can conclude that not every region will be associated with a substorm.

### 4.2 THEMIS DFB List

The second validation method used a list of DFB arrivals measured by THEMIS. The DFB list covers 2013–2015 when there were three THEMIS satellites in the inner magnetosphere with apogees of ∼12*R*_*E*_. The full comparison table is in Table S5. For each DFB time in the list, *t*_0_, we searched for TWINS intervals in a window from *t*_0_ − 20 min to *t*_0_ + 20 min for an identified region. Results of the modified binary analysis are shown in Table 2 with the same format as Table 1. Note that these values are different from those in the parameter search in Table S1 due to additional changes in other parameters following this comparison, including the smoothing window (Step 0) and the max temperature value (Step 2). The results indicate that the algorithm is highly likely to identify a region in the TWINS data when there is a DFB. We did not consider the likelihood of a detected DFB for all identified TWINS intervals due to the limitations of the satellite locations.

**Table 2:** Modified Binary Analysis of Two Wide‐Angle Imaging Neutral‐Atom Spectrometers Algorithm Compared to THEMIS Dipolarizing Flux Bundle List

	All data	qf ≥ 1	qf ≥ 2	qf ≥ 3	qf ≥ 4	qf = 5
TWINS avail	63	60	60	56	55	50
TWINS found	56	53	53	49	49	45
TWINS missed	7	7	7	7	6	5
% missed	11%	12%	12%	13%	11%	10%
% found	89%	88%	88%	88%	89%	90%

**Figure 2:**
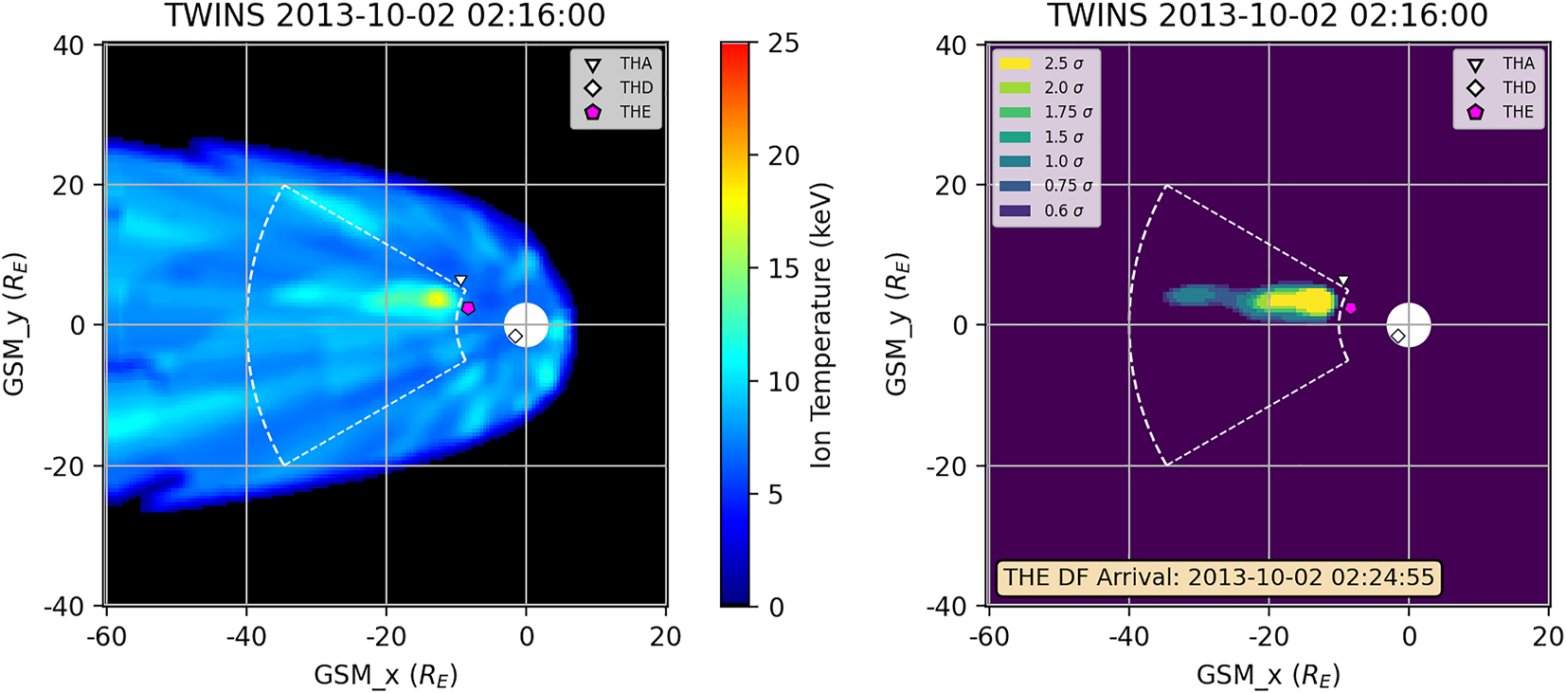
(left) Ion temperature map derived from Two Wide‐angle Imaging Neutral‐atom Spectrometers for 2 October 2013 at 02:16 UT. (right) *Z*‐score map of identified region. Same format as Figure 1. Locations of THEMIS satellites shown by symbols, with magenta indicating the satellite that measured the dipolarizing flux bundle (DFB). A DFB arrival was measured by THEMIS E at 02:24:55 UT as indicated in the right panel.

A sample interval with DFB arrival and algorithm‐identified region is shown in Figure 2. For this interval, the location of THEMIS in the map is identified by using the *x*‐ and *y*‐ coordinates of the satellite location. We also tried a projection method that takes into account the distance of the THEMIS satellite from the *x*‐*y* plane that resulted in locations within two grid bins (about half the size of the symbols). The identified region does not overlap with the location of the THEMIS satellite at the time of the DFB measurement, indicating that the most Earthward component of the DFB (i.e., the DF) is not included in the identified region. Even in the temperature map (left panel), the THEMIS satellites do not appear within the region of increased ion temperatures. Upon reviewing all of the intervals with both identified regions and DFB measurements, this is typically the case, but appears to be in part due to the orbital limitations of the THEMIS satellites as well as the inner boundary of the defined search area.

## 5 Case Studies

Prior to having the algorithm, intervals of interest were found in TWINS data either by identifying interesting intervals from in situ measurements or by looking manually through analyzed TWINS data. As an example of the former method, the 3 August 2016 interval mentioned previously and analyzed in detail in Keesee et al. (2021) was selected by a flow reversal identified in MMS data. That date had a very modest storm (−52 nT minimum Dst, Kyoto database) that could have been overlooked. In contrast, using the latter method gives us the opportunity to take advantage of the global coverage of the TWINS mission to identify interesting intervals even when in situ measurements are not available. Here we present two intervals identified by the algorithm and validated as having significant magnetotail activity by DFB detections with THEMIS. Having these identified and validated intervals will enable us to conduct in‐depth studies of these events and compare with other TWINS maps to improve our understanding of the phenomena observed by TWINS. The first such interval was presented in Figure 2. This interval is interesting because THEMIS A did not detect a DFB, despite being near but toward the side of the region of enhanced ion temperatures. This interval will be useful for studying the spatial extent of the DFBs.

Figure S2 in Supporting Information S1 shows an interval on 4 April 2010 at 11:45 UT during which some minor activity was driven by increased solar wind speeds (NOAA Weekly, 2010‐prf1805). The Dst index ranged from −11 to −25 nT and there were intervals of elevated AE index. As seen in Figure S2 in Supporting Information S1, the THEMIS satellites were located close together such that their symbols overlap and a DFB was measured at 11:25 UT by both THEMIS D and E, before the TWINS interval, and another measurement was recorded by THEMIS E at 11:48 UT, after the TWINS interval. This case can help us consider timing of movement of these phenomena. There is also a small region in the temperature map that may indicate a separate structure that results in the first DFB measurement.

## 6 Algorithm Application to Simulation Output

**Figure 3:**
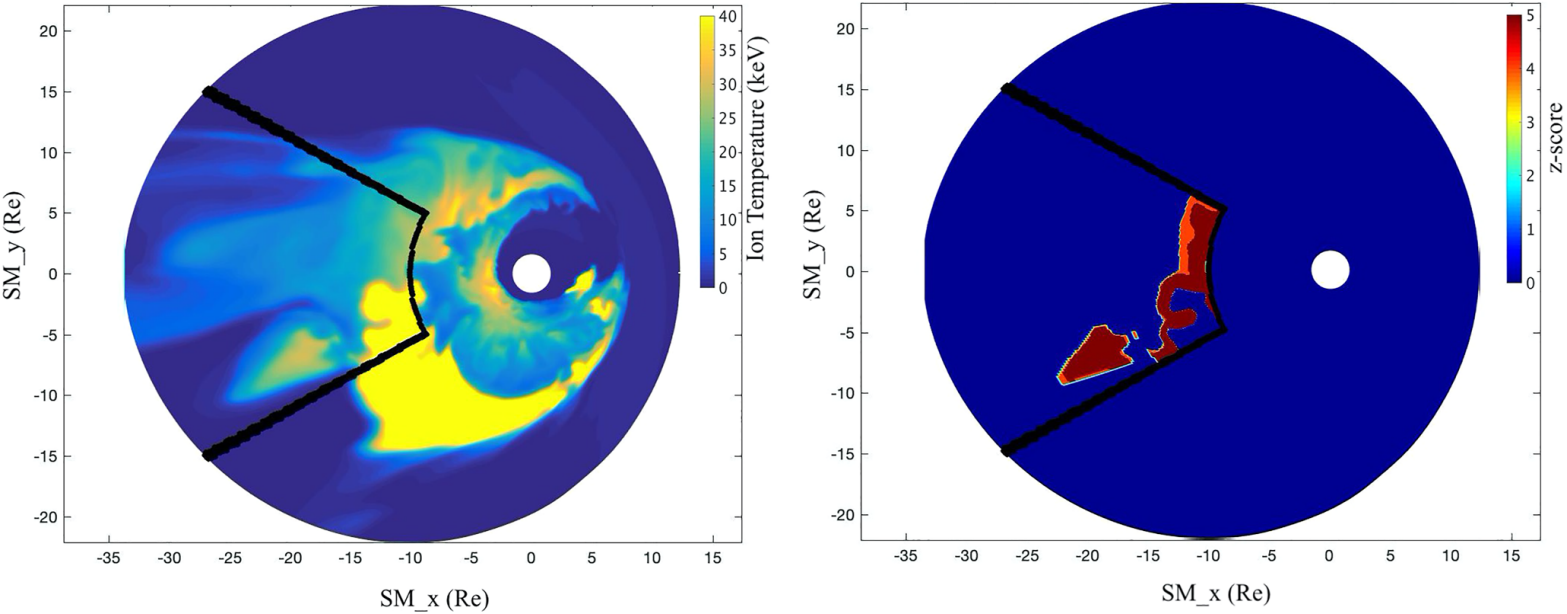
(left) Ion temperature from MAGE simulation. Black outline indicates algorithm search area. (right) *Z*‐score map of identified regions.

To demonstrate the potential application of the algorithm to other datasets, we ran a preliminary test on simulation output. The simulation data are generated using the Multiscale Atmosphere Geospace Environment (MAGE) model (Lin et al., 2021; Pham et al., 2022) and correspond to the main phase of the 17 March 2013 geomagnetic storm (Sorathia, ohtani, et al., 2021; Sorathia, Michael, et al., 2021). For this test we removed the smoothing (Step 0) and mapped the curvilinear grid to the *x*‐*y* plane, but otherwise did not adjust any parameters in the algorithm. A sample interval is shown in Figure 3. (Note that the cutout of the identified region occurs because of the maximum temperature set in Step 2, which was not changed for this test). This demonstrates that the algorithm could be used to compare such regions observed in data and models for comparative studies and validation, enabling a new method of mesoscale studies. The algorithm could be applied to different simulation output parameters to understand various properties of these mesoscale features.

## 7 Summary and Discussion

We have developed an algorithm to identify intervals with regions of increased ion temperatures in ion temperature maps calculated from TWINS ENA data. We believe that these regions are associated with mesoscale phenomena in the magnetotail known as flow channels and DFB. We note that varying the algorithm parameters makes quite a difference in selection of intervals. We tried to include intervals with known observations of flow channels from past studies without over‐selecting intervals that could lead to skewed results, particularly for statistical studies. While this sensitivity to input parameters may have implications for underlying physics, it could also be due to variations in overall ENA flux. The ENA flux measured is dependent on the neutral density in the region of the parent ion population, which varies quite a bit with location and geomagnetic activity (e.g., McComas et al., 2002; Zoennchen et al., 2015). These variations will be considered in future studies.

We have presented a validation of the method using both a database of substorm onsets and a list of DFB measurements. It is interesting to consider the time variation in the measurements between the two datasets. For the substorm onset comparison, we can see from Table S2 in Supporting Information S1 that regions of increased ion temperature are observed both before and after the substorm onset time for every interval where TWINS data were available for the full time window (twenty‐one time windows with full data, three without). Recall that the search window is *t*_0_ − 15 min to *t*_0_ + 30 min around the TWINS interval time. The average duration from the first to last TWINS interval for which the SuperMAG onset time falls within that window is 30 min. The average time of onset is 21 min after the first interval. This indicates that observations are made only toward the end of the substorm growth phase and most of the expansion phase, based on average durations of these phases given by Forsyth et al. (2015).

The algorithm will enable future statistical and event studies, and we have presented some potential examples. In particular, we will conduct case studies on the intervals described in Section 5 to understand the spatial extent and timing of these phenomena. We are running the algorithm on our database of 10‐min cadence temperature maps (Keesee et al., 2020) and will present statistics of the regions identified. We will also run the algorithm on MAGE simulation output to conduct data‐model comparisons and validation as described in Section 6.

## Supporting information

Supporting Information S1

Table S1

Table S2

Table S3

Table S4

Table S5

## Data Availability

Two Wide‐angle Imaging Neutral‐atom Spectrometers (TWINS) data and the database of TWINS ion temperature maps are available on CDAWeb (https://cdaweb.gsfc.nasa.gov). The IDL scripts to create ion temperature maps from TWINS data are available at Keesee et al. (2019).
