# Supplementary material for: Automated Detection Algorithm for Mesoscale Heated Regions in TWINS Ion Temperature Maps
Source: J Geophys Res Space Phys. 2022 Sep 23;127(9):e2022JA030464. doi: 10.1029/2022JA030464 (PMC9788308; doi:10.1029/2022JA030464)
Supplement: Supplementary file 1 — Supporting Information S1 [file JGRA-127-e2022JA030464-s004.docx]

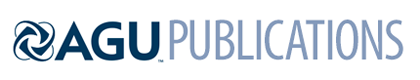


*Journal of Geophysical Research Space Physics*

Supporting Information for

**Automated detection**

**algorithm for mesoscale heated regions in TWINS**

**ion temperature maps**

A. M. Keesee^1,2^, R. Katus^3^, J. Tibbetts^1^,J. Liu^4^, X. Zhang^4^, K. A. Sorathia^5^

^1^Department of Physics and Astronomy, University of New Hampshire, Durham, NH, USA
^2^Space Science Center, University of New Hampshire, Durham, NH, USA
^3^Department of Mathematics, Eastern Michigan University, Ypsilanti, MI, USA
^4^Department of Earth, Planetary, and Space Sciences and Institute of Geophysics and Planetary Physics, University of California, Los Angeles, CA, USA
^5^The Johns Hopkins University Applied Physics Laboratory, Laurel, MD, USA

**Contents of this file**

Figures S1 to S2

Table S2

**Additional Supporting Information (Files uploaded separately)**

Captions for Tables S1 and S3 to S5

**Introduction**

The Supplemental Information includes the five tables used for the validation of the algorithm and two figures with algorithm output described in the main paper.


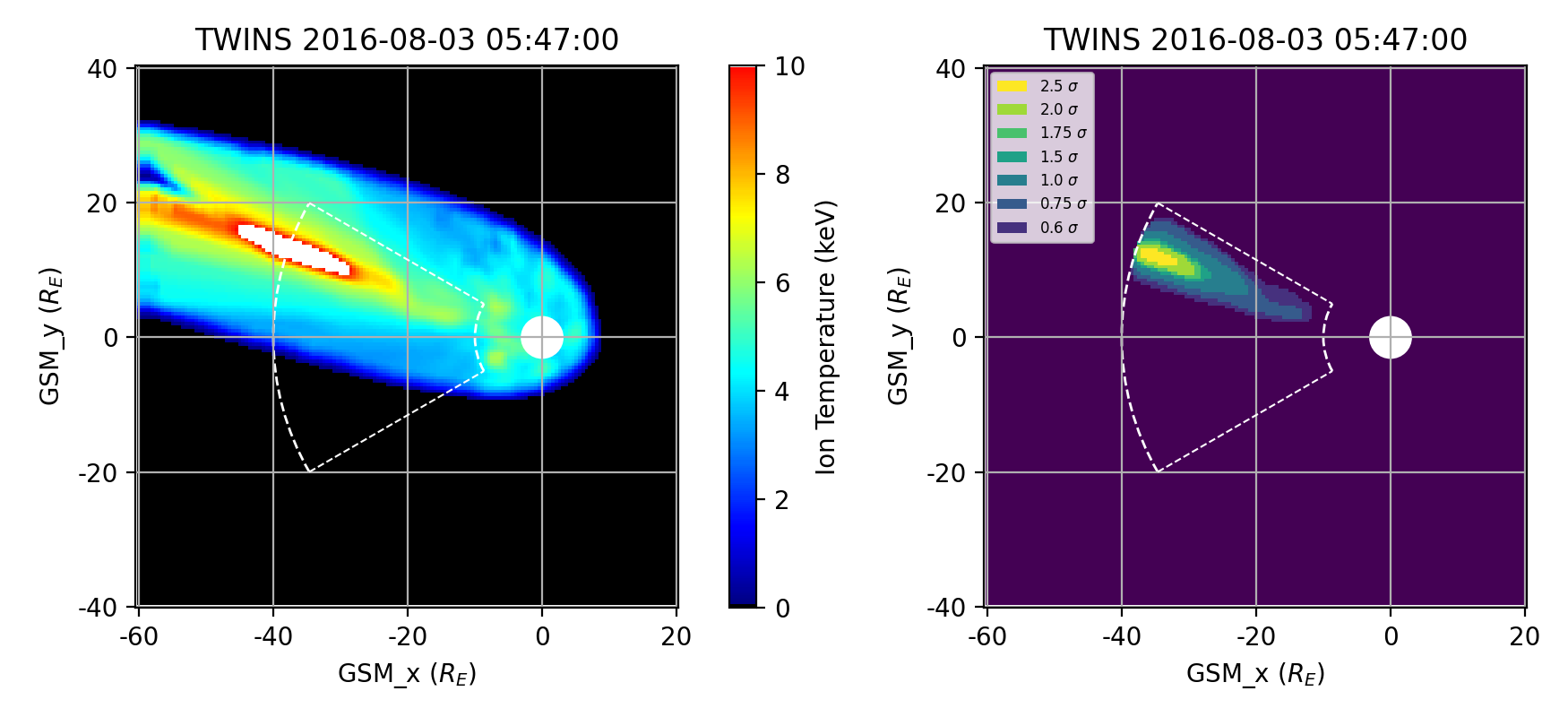


Figure S1. (left) Ion temperature map derived from TWINS for August 3, 2016 at 05:47 UT. (right) Z-score map of identified region. Same format as Figure 1 of the main text.


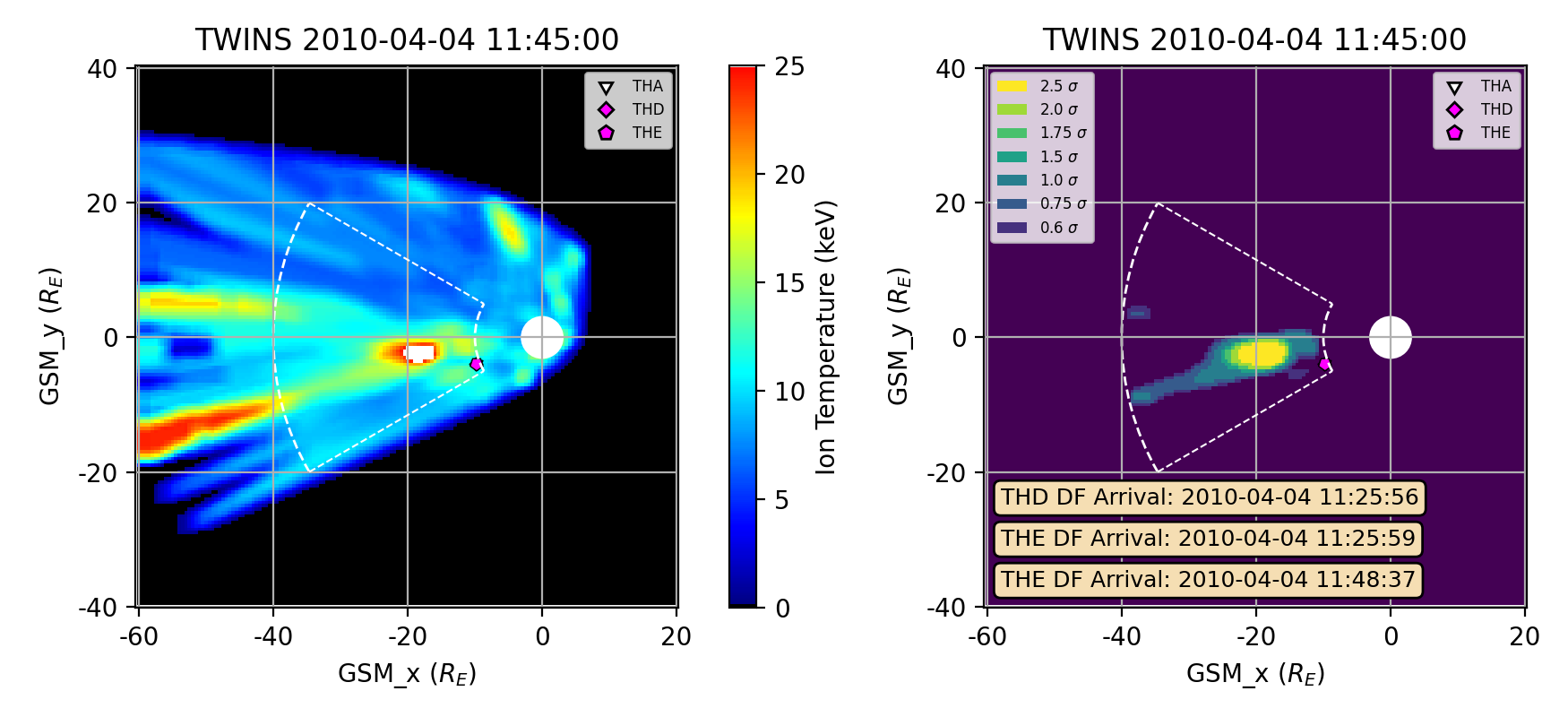


Figure S2. (left) Ion temperature map derived from TWINS for 4 April 2010 at 11:45 UT. (right) Z-score map of identified region. Same format as Figure 2 of the main text.

Table S1. Parameter comparison for limits of r and z-score threshold using modified binary analysis with DFB list

Table S2. List of storms used for SuperMAG comparison and minimum Dst value on that date (Kyoto Dst database)

| Date | Minimum Dst (nT) |
| --- | --- |
| 2-Oct-2013 | -72 |
| 12-Apr-2014 | -87 |
| 27-Aug-2014 | -79 |
| 10-11-Apr-2015 | -51, -75 (double peak) |
| 7-Sep-2015 | -70 |
| 20-Sep-2015 | -75 |
| 4-Nov-2015 | -60 |
| 8-May-2016 | -88 |
| 3-Aug-2016 | -52 |
| 23-Aug-2016 | -73 |
| 21-Dec-2016 | -40 |

Table S3. Comparison of substorm onset list with TWINS intervals identified by the algorithm that occurred within the time frame including 30 minutes before and 15 minutes after onset time. Columns included are:

- SM Substorm Onset: Date and time (UTC) of substorm onset from SuperMAG website using (Forsyth et al., 2015) method
- SM_mlt: Magnetic local time of substorm onset
- SM_mlat: Magnetic longitude of substorm onset
- TWINS_QF_avg: average data coverage quality flag for all TWINS intervals within the time frame
- TWINS_rhomin: minimum ρ value (distance from Earth) of identified region in TWINS map
- TWINS_rhomax: maximum ρ value (distance from Earth) of identified region in TWINS map
- TWINS_mlt: Magnetic local time of center of identified region in TWINS map
- TWINS_regioncount_avg: number of grid bins in the regions identified by the algorithm (≧0.6 σ) averaged for all TWINS intervals within the time frame

Table S4. Comparison of TWINS intervals identified by the algorithm with substorm onsets occurring within the time frame including 15 minutes before and 30 minutes after the TWINS interval time. Columns included are:

- TWINS Interval: Date and time (UTC) of TWINS interval
- SM Substorm Onset: Date and time (UTC) of substorm onset occurring within the time frame. If no onset occurred within the time frame, the value is listed as 00-Jan-0000 00:00:00.
- TWINS_SuperMag: Set to 1 if an onset occurred within the time frame, 0 otherwise.
- SYMH: Sym-H index (nT) from OMNIWeb
- Dst: Dst index (nT) from OMNIWeb
- Ae: AE index (nT) from OMNIWeb
- TWINS_QF: data coverage quality flag
- TWINS_rhomin: minimum ρ value (distance from Earth) of identified region in TWINS map
- TWINS_rhomax: maximum ρ value (distance from Earth) of identified region in TWINS map
- TWINS_MLTmid: Magnetic local time of center of identified region in TWINS map
- TWINS_RegionCount: number of grid bins in the regions identified by the algorithm (≧ 0.6 σ)
- SM_mlt: Magnetic local time of substorm onset
- SM_mlat: Magnetic longitude of substorm onset

Table S5. Comparison of THEMIS dipolarizing flux bundle (DFB) measurements with TWINS intervals identified by the algorithm that occurred within the time frame including 20 minutes before and 20 minutes after the DFB arrival time. Note that the DFB event list in the table is a subset of that in Zhang et al., (2019), so see Liu et al., (2013) for more detailed descriptions and please also cite those references when using the list. GSM coordinates used throughout. Columns included are:

- DFBarrivalTime: Date and time (UTC) of DFB arrival measured by THEMIS
- spacecraft_name: which THEMIS spacecraft made the DFB measurement
- TimeOfBzMin: Date and time (UTC) of minimum magnetic field z-component
- TimeOfBzMax: Date and time (UTC) of maximum magnetic field z-component
- nx_frontnormal: Normal vector to the dipolarization front, x-component (The front normal vector is determined from B_in_ × B_out_ where B_in_ and B_out_ are the magnetic field in the direction of the inner and outer boundary of the dipolarization front, respectively.)
- ny_frontnormal: Normal vector to the dipolarization front, y-component
- nz_frontnormal: Normal vector to the dipolarization front, z-component
- FrontThetaAngle: Angle between B_in_ and B_out_
- vx: Velocity of the front, x-component (km/s)
- vy: Velocity of the front, y-component (km/s)
- vz: Velocity of the front, z-component (km/s)
- GSMX: Orbital location of the THEMIS satellite, x-component (R_E_)
- GSMY: Orbital location of the THEMIS satellite, y-component (R_E_)
- GSMZ: Orbital location of the THEMIS satellite, z-component (R_E_)
- TWINSDataAvail: Set to 1 if there is a TWINS map available in the time frame, 0 otherwise
- TWINSactivity: Set to 1 if the algorithm identifies a region in the time frame, 0 otherwise
- TWINSRegionCount: number of grid bins in the regions identified by the algorithm (≧ 0.6 σ)
- TWINSQuality: data coverage quality flag
- TWINSrhomin: minimum ρ value (distance from Earth) of identified region in TWINS map
- TWINSrhomax: maximum ρ value (distance from Earth) of identified region in TWINS map
- TWINSMLTmin: minimum magnetic local time of identified region in TWINS map
- TWINSMLTmax: maximum magnetic local time of identified region in TWINS map
